# What are the underlying reasons behind socioeconomic differences in doctor‐patient communication in head and neck oncology review clinics?

**DOI:** 10.1111/hex.13163

**Published:** 2020-11-23

**Authors:** Sarah Allen, Simon N. Rogers, Steven Brown, Rebecca V. Harris

**Affiliations:** ^1^ Department of Health Services Research Institute of Population Health Sciences University of Liverpool Liverpool UK; ^2^ Evidence‐Based Practice Research Centre (EPRC) Faculty of Health and Social Care Edge Hill University Ormskirk UK; ^3^ Consultant Regional Maxillofacial Unit University Hospital Aintree Liverpool UK; ^4^ Department of Psychological Sciences Institute of Population Health Sciences Liverpool UK; ^5^ Department of Health Services Research Institute of Population Health Sciences Liverpool UK

**Keywords:** communication, doctor‐patient relationship, inequalities, patient participation, socioeconomic status

## Abstract

**Objective:**

To explore socioeconomic status (SES) differences in patterns of doctor‐patient communication within head and neck cancer clinics and why such differences exist.

**Methods:**

Thirty‐six head and neck cancer review appointments with five Physicians were observed and audio‐taped, along with follow‐up interviews involving 32 patients. Data were analysed using Thematic Analysis, and compared by patient SES (education, occupation and Indices of Multiple Deprivation).

**Results:**

Three main themes were identified: (a) Physicians used more humour and small talk in their consultations with high SES patients; (b) Low SES patients were more passive in their participation, engaged in less agenda setting and information‐seeking, and framed their clinical experience differently; (c) Low SES patients had different preferences for involvement, defining involvement differently to high SES patients and were seen to take a more stoical approach.

**Conclusion:**

Low SES patients take a more passive role in medical consultations, engage in less relational talk and are less likely to raise concerns, but were satisfied with this. Physicians may adapt their communication behaviour in response to low SES patients’ expectations and preferences.

**Practice Implications:**

A question prompt list may help low SES patients to raise concerns during their consultations. This may reduce inequalities in communication and health.

## 1 INTRODUCTION

Effective doctor‐patient communication contributes to better treatment outcomes.^1^, ^2^, ^3^, ^4^, ^5^ Its importance makes it the ‘heart and art’ of medicine.^6^ Good doctor‐patient communication is characterized by a two‐way dialogue between patient and doctor, rather than a situation where one party dominates the conversation.^7^ Effective communication is therefore seen where patients actively participate in consultations by asking questions and expressing opinions and concerns; and doctors involve patients by encouraging them to ask questions and express opinions or preferences.^7^ Such input from patients is necessary in order for shared decision‐making to occur.^7^

Studies show that there are socioeconomic status (SES) differences in doctor‐patient communication. Patients from low SES backgrounds tend to participate less actively in their consultations by asking fewer questions and are less likely to volunteer information unprompted or express their opinions or emotions.^8^, ^9^ Observations also show that clinicians give less information, spend less time building rapport, and listen less attentively to these patients.^8^, ^9^, ^10^, ^11^ Thus, a patient's socioeconomic position affects both the way in which they communicate with doctors, as well as how doctors communicate with them. This is likely to contribute to socioeconomic inequalities in health.

There are a number of theories which have been proposed to explain these socioeconomic differences in doctor‐patient communication. Status characteristics theory posits that patients’ and doctors’ perceived differences in social status influence how much they participate in the consultation, and the value placed on their contributions. Status is determined by characteristics such as expertise, qualifications, SES, ethnicity and gender. The higher an individual's status, the more opportunities they are given to participate and the more their participation is valued.^12^, ^13^ Thus, patients who believe their status to be low may deny themselves participation, and doctors perhaps unconsciously deny participation to patients of lower status. Lay theories of social class provide a slightly different perspective. Low SES individuals who believe their social status is biologically determined and unchangeable may experience more shame, anxiety and negative affect than those who do not because of fatalism, thus reducing participation in consultations.^14^

However, there has been a lack of research to explain these socioeconomic differences. Furthermore, the impact of SES on the effectiveness of the doctor‐patient relationship and communication in head and neck cancer has not been well studied.^15^ Malignancies of the head and neck are the 8th most common cancer in the United Kingdom; approximately 4000 die annually from the disease.^16^ Previous work has demonstrated an association between low SES and unfavourable health‐related quality of life outcomes after a diagnosis of head and neck cancer,^17^, ^18^ although there has been little attention in the doctor‐patient communication literature. To fill this knowledge gap, the objective of this study was to explore differences in doctor‐patient communication practices based on patient SES and why any differences exist using a qualitative design.

## 2 METHODS

The setting for the study was head and neck oncology review clinics. After obtaining ethics board approval, patients over the age of 18 years who had previously completed treatment for cancer of the head or neck during the previous 12‐24 months were recruited for study participation from one of five specialty clinics at a tertiary care hospital in the North‐West of England. Each of these clinics was run by male physicians, and 36 patients were recruited in total. Non‐English‐speaking patients, those unable to provide informed consent, and those whose SES could not be determined were excluded.

Patients were approached to take part in the study while waiting for their consultation. Informed consent about the study was taken. A researcher observed and audio‐taped the patient's head and neck review appointment with their Physician, sitting in the corner of the room. The researcher's field notes were kept to record phenomena not available to audio recording. In particular, notes were made on body language such as eye contact, whether the patient and Physician shook hands, facial expressions, etc Appointment length was timed from introduction to closing statements by either patient or surgeon. A semi‐structured interview with the patient was arranged a few days later, which was undertaken by telephone or face‐to‐face. The consultation recordings were used to derive questions for each follow‐up interview and personalize the topic guide, so that the interviewer could ask each patient about specific things which happened during their appointment (eg, You mentioned you were having difficulty swallowing, how did you feel about the physician's response to that?). The researcher engaged in specific training in qualitative observation and coding at formal workshops and though private study.

Thirty‐two patients participated in post‐consultation interviews. Three patients could not be contacted by the researcher following the appointment, despite repeated attempts, and one patient declined to be interviewed following their consultation. There were consequently 68 data sources (36 appointments and 32 linked interviews). Recruitment was stopped when a spread of participants by SES characteristics was achieved, and when saturation of data was reached.

We collected multiple datapoints on each patient to describe SES, including indices of multiple deprivation (IMD) based on postcodes, patient occupation mapped to the Office for National Statistics standard occupational classifications (SOC), and patient‐reported education data and employment status. IMD decile one represents the most deprived postcodes.^19^ SOC is a commonly used classification of occupational data which is collected and routinely updated by the UK government, and has been used as an SES measure by a number of studies.^20^, ^21^, ^22^ It consists of nine groups, with group 9 representing the lowest SES.^23^, ^24^ These data were aggregated for each patient, as available, to categorize study subjects into ‘low’ or ‘high’ SES. Details regarding patient SES categorization are available in Table 2.

Audio recordings of appointments and interviews were transcribed and, with fieldnotes, organized using the NVivo 10 software, analysed using thematic analysis. This analysis involved identifying common conversational characteristics that were combined into themes and sub‐themes, which together addressed the research questions. Differences by SES background were identified using a process of constant comparison between low SES and high SES cases. This involved seeking out contradictions in themes and sub‐themes as cases were added to the analysis, and amending themes until adding new cases to the analysis did not result in any reframing of the emergent themes. A researcher (SA) undertook initial coding and generated an initial thematic framework which was refined following discussion with other authors (RH, SB). Analysis took place alongside data collection. Each patient was assigned a number to anonymize the data. These codes used are to denote participants in the results below.

## 3 RESULTS

### 3.1 Sample characteristics

Although 36% of participants lived in IMD decile 1, there was also a spread of participants across the SES gradient. The majority of participants were men (n = 24, 66.7%), with the most common primary treatment being surgery alone (n = 13, 36.1%), closely followed by chemoradiotherapy alone (n = 10, 27.8%). Average age was 64.9 years (Table 1). Table 2 shows the distribution of patients across SES categories.

### 3.2 Overview

**Table 1:** Patient characteristics

Characteristics	Total (N = 36) N (%)
Gender	
Male	24 (66.7%)
Female	12 (33.3%)
Mean age	64.9 years (SD = 11.30)
Mean appointment length (minutes: seconds)	8:03 (SD = 3:56)
Indices of Multiple Deprivation (IMD) decile	
1 (most deprived)	13 (36.1%)
2	2 (5.6%)
3	3 (8.3%)
4	5 (13.9%)
5	2 (5.6%)
6	2 (5.6%)
7	2 (5.6%)
8	2 (5.6%)
9	4 (11.1%)
10 (least deprived)	1 (2.8%)
Mean age leaving education (Information not available, n = 4)	16.9 years (SD = 3.80)
Highest qualification	
None	14 (38.9%)
GCSE or equivalent	10 (27.8%)
Advanced level (A level) or equivalent	3 (8.3%)
Undergraduate	3 (8.3%)
Postgraduate	2 (5.6%)
Information not available	4 (11.1%)
Office for National Statistics (ONS) group	
1 (highest)	5 (13.9%)
2	3 (8.3%)
3	2 (5.6%)
4	2 (5.6%)
5	5 (13.9%)
6	7 (19.4%)
7	2 (5.6%)
8	6 (16.7%)
9 (lowest)	0 (0%)
Information not available	4 (11.1%)
Employment status	
Employed	9 (25%)
Retired	21 (58.3%)
Sick leave	1 (2.8%)
Unemployed	1 (2.8%)
Information not available	4 (11.1%)
Primary treatment	
Surgery	13 (36.1%)
Radiotherapy	6 (16.7%)
Chemoradiotherapy	10 (27.8%)
Surgery with radiotherapy	6 (16.7%)
Surgery with chemoradiotherapy	1 (2.8%)

**Table 2:** Patient SES

Patient	IMD decile	Age leaving formal education	Highest qualification	ONS major group	Employment status	Overall SES
1	9	35	Postgraduate	6	Employed	High SES education, low SES occupation
2	2	NA	NA	NA	NA	Insufficient information
3	3	16	Undergraduate	7	Employed	High SES education, low SES occupation
4	1	15	None	6	Retired	Low SES both domains
5	8	16	GCSE or equivalent	7	Retired	Low SES both domains
6	1	15	None	5	Sick leave	Low SES both domains
7	3	15	None	6	Retired	Low SES both domains
8	4	16	GCSE or equivalent	5	Employed	Low SES both domains
9	9	16	Undergraduate	1	Retired	High SES both domains
10	1	16	GCSE or equivalent	3	Retired	Low SES education, high SES occupation
11	1	15	GCSE or equivalent	4	Retired	Low SES education, high SES occupation
12	4	22	Undergraduate	2	Retired	High SES both domains
13	4	15	None	5	Retired	Low SES both domains
14	1	16	None	4	Employed	Low SES education, high SES occupation
15	4	15	None	8	Retired	Low SES both domains
16	9	20	A level or equivalent	5	Employed	High SES education, low SES occupation
17	5	15	None	1	Retired	Low SES education, high SES occupation
18	1	16	GCSE or equivalent	6	Unemployed	Low SES both domains
19	10	15	None	8	Retired	Low SES both domains
20	2	17	A level or equivalent	8	Employed	High SES education, low SES occupation
21	1	15	None	6	Retired	Low SES both domains
22	7	*NA*	*NA*	*NA*	*NA*	*Insufficient information*
23	6	16	GCSE or equivalent	1	Employed	Low SES education, high SES occupation
24	1	16	GCSE or equivalent	8	Employed	Low SES both domains
25	6	16	GCSE or equivalent	1	Retired	Low SES education, high SES occupation
26	1	17	None	6	Retired	Low SES both domains
27	1	NA	NA	NA	NA	Insufficient information
28	1	NA	NA	NA	NA	Insufficient information
29	1	20	A level or equivalent	5	Employed	High SES education, low SES occupation
30	5	16	GCSE or equivalent	2	Retired	Low SES education, high SES occupation
31	9	22	Postgraduate	2	Retired	High SES both domains
32	7	17	None	1	Retired	Low SES education, high SES occupation
33	4	15	None	3	Retired	Low SES education, high SES occupation
34	1	15	None	8	Retired	Low SES both domains
35	3	15	None	8	Retired	Low SES both domains
36	8	15	GCSE or equivalent	6	Retired	Low SES both domains

We identified three main differences by SES in patterns of communication, which were sub‐divided into seven sub‐themes in all (Table 3). We noted that consultations differed by SES in firstly the extent of relational talk; secondly in the extent to which patients approached the consultation taking either an active or a passive role; and thirdly in the preferences (both expressed and demonstrated) of patients for information about their condition, and the extent of involvement in decisions about their care.

**Table 3:** Overview of themes and sub‐themes

Main themes	Sub‐themes
Relational talk (Talk used to develop interpersonal relations between clinician and patient)	The use of humour (High SES patients were more likely to make jokes, banter and sarcastic comments during conversation in the appointment)
	Small talk (High SES patients were more likely to engage in more lengthy polite conversation not relating to the main purpose of the appointment)
Active or passive participation (in the consultation by patients)	Education and occupation as a cultural frame of reference
	Patients with an agenda (High SES patients came to their appointments with a list of things to discuss)
	Responsibility for obtaining information (High SES patients felt it was their responsibility to get information)
Preferences for involvement (in conversation and decision‐making during the consultation)	Defining involvement in decision‐making (High SES prefer knowing and being involved in determining the which treatment options are taken up)
	Stoicism (Low SES patients had a more stoic attitude)

### 3.3 Relational talk

#### 3.3.1 The use of humour

We observed that the tone of consultations was warmer and more familiar when patients from higher SES groups were seen. Doctors were friendly and talkative towards patients in higher occupational classes, spending time to recognize and build rapport in the relationship between the two. The use of humour in these interactions was particularly characteristic, with banter usually initiated by the doctor. In Table 4 (Theme 1, sub‐theme 1.2), we give an example of banter during the farewell sequence involving higher SES Patient 25, which contrasts with a much more formal, and shorter appointment ending with lower SES Patient 6. A contrast in formality is also seen here, where the Physician addresses Patient 6 as ‘Mr.’, but Patient 25 as ‘mate’, following their ‘joke’.

**Table 4:** Contrast between high and low SES patients in communication content

Themes and sub‐themes	Patients with high socioeconomic backgrounds	Patients with low socioeconomic backgrounds
1. Relational talk		
1.1 Relational talk in the opening sequence	Physician: ‘No problems’? Patient: ‘No I feel great now. I feel back to normal’. Physician: ‘Yes? Still doing charity races and things like that’? (eye contact, sat closer) Patient: ‘I did erm… I did a hike for Macmillan Cancer in June… so I am minus about 6 toe‐nails at the moment because it's a marathon hike in the Lake District. So it took 14 hours’. Physician: ‘Oh right. Not the right shoes and all that’? Patient: ‘No… I had, it was just, you know, the terrain’. Patient 23, Physician 1, Consultation	Physician: ‘How are you’? Patient: ‘Fine’. Physician: ‘Good. No problems’? Patient: ‘No none at all’. Physician: ‘That's what we like to hear. Shall we have a little look down’? Patient: ‘Yeah yeah’. Patient 18, Physician 3, Consultation
1.2 Relational talk in the farewell sequence	Patient: ‘Thank you very much’ (shake hands, both sitting). Physician: ‘So see you in 3 months’. Patient: ‘Alright, thank you’. Physician: ‘Yeah, so for the person recording – he is very grateful he said!!!’ Patient: ‘I am extremely grateful’. Physician: ‘I know you are’. Patient: ‘Superb expertise’. Physician: ‘Cheers mate. I know you mean that by the way. Thank you very much’. Patient: ‘Thank you’. Patient 25, Physician 1, Consultation	Physician: ‘Alright Mr (patient)….. I can't see anything (Facing patient, making eye contact). We'll just get the scans to look at the tissues inside. I think, I think it's just the result of the surgery of the radiotherapy causing scarring in there, but we will get the scan and it will also act as a baseline for us. Now, I’ll see you back on the 7th June.’ Patient: ‘Alright, thanks very much’. Physician: ‘Alright, take care now. (shake hands both sat down, Physician lifts up a bit off his chair). Bye’. Patient: ‘Bye’. Patient 6, Physician 2, Consultation
2. Active or passive participation		
2.1 Education and occupation as a cultural frame of reference	Patient: ‘I know a lot of patients going in they are probably quite nervous when they go to see a Physician like, you know what I mean, or any doctor, not just because of the illness but because of they see them as somebody quite powerful and very professional and very different. But having worked in that environment over the years I can converse with them much easier. I know the system I know how the system works and make it easier for myself and them as well’. Patient 12, Physician 3, Interview	Patient: ‘See I am in the motor trade as an auto‐electrician and erm, mechanics, and so I am mechanically minded. So, so, I know if someone is saying something is right, then it's got longevity at least’. Patient 8, Physician 1, Interview
2.2 Patients with an agenda	Patient: ‘Any concerns and you can bring anything up, I don't feel it's going to be a stupid question.” Interviewer: “Yeah. So, there wasn't anything else which you wanted to ask but didn't get out’? Patient: ‘No, I generally have a question every time I go…(chuckles)…I generally bring something up’. Interviewer: ‘You seem very comfortable to ask the questions you want’. Patient: ‘Yeah, Yeah I do, as I say in the past usually at every consultation that I have had I have asked something about some part of my mouth, throat, tongue…’ Patient 3, Physician 2, Interview	Interviewer: ‘You mentioned your problems with swallowing to Mr (Physician) in the appointment’? Patient: ‘I didn't I thought I would wait for the outcome, get the camera down and I will wait for the outcome and then if it was that the cancer had progressed then I would tell him how I felt about it beforehand’. Interviewer: ‘Yeah’. Patient: ‘But I didn't mention it’. Interviewer: ‘Okay, I think erm…you mentioned it a bit after he had given you the kind of…’ Patient: ‘He said ‘All clear’ and I said ‘Thank goodness for that’.’ Interviewer: Laughs… Patient: ‘That's all I said. I didn't say I had been worried or anything’. Patient 7, Physician 1, Interview
2.3 Responsibility for obtaining information	Patient: ‘Yeah…occasions erm…I have phoned up and said look I forgot to ask this or forgot to ask that, erm and I have done that with my doctor as well and someone phones me back or I phone back when they tell me to phone back or I get a letter or whatever. I think if you ask you will get the information’. Patient 3, Physician 2, Interview	Interviewer: ‘Do you feel like you get enough information from them’? Patient: ‘Yes yeah’. Interviewer: ‘Erm and do you find that you get reassurance as well’? Patient: ‘Yeah yeah. I mean especially with Mr (Physician), I mean he is straight with you’? Interviewer: ‘Yeah’. Patient: ‘I mean he won't try and kid you or nothing’ Interviewer: ‘Mmm’ Patient: ‘If he thinks there is something wrong he tells you he thinks there is something wrong’. Interviewer: ‘Yeah’ Patient 6, Physician 2, Interview
3. Preferences for involvement		
3.1 Defining involvement in decision‐making	Patient: ‘Yeah I like to be involved ‘cause I like to know what is going on. Erm…I have always been explained to me why they are doing things and initially when the treatment was. When I was diagnosed and they said to me, “Well there is two courses of treatment,” he said “There is the tried and tested one or there is a new erm…one that they are trialling”… But obviously its not as, they do not know the results, so he said: “What do you want to do?”, he said “It's got to be your decision”.’ Patient 3, Physician 2, Interview	Patient: ‘Erm…well I expect to be 100% involved…You know if, say he was going to operate on me or things like that…I would like to be involved. You know I would want to know everything about it before it actually happened…And like I say I have only had the one operation you know when he took the tumours out and he explained everything about that you know before it got done and then after it had been done, the first time I seen him afterwards…He went through everything with me, he explained it all. He was very good, he really is.’ Patient 6, Physician 2, Interview
3.2 Stoicism	Patient: ‘I am comfortable like about expressing things, like you know what I mean like. I like to talk things through, I like to get to the very bottom of things. I like to get to understand it myself, like you know, because of my science, medical background I like to know and understand anything, you know what I mean? If there's something I don't know about, tell me more about it, I want to know’. Patient 12, Physician 3, Interview	Patient: ‘But I keep it to myself, I don't say it to my daughter as I don't like to upset her, you know, worry her. I wouldn't like to worry her and she says to me’ ‘Have you got any pains? Don't forget to tell the doctors, tell them everything, write everything down.’ I say, ‘I’m Okay, Okay’. I just keep on saying to myself that it's the chemo or the radio because a lot happens to the inside of your body so this is why this is happening and things like that and thinking about everything that he says to you and I just… you just have to get on with it’. Patient 13, Physician 4, Interview

In total, there were 103 ‘jokes’ between patients and Physicians across 32 observed appointments (excluding the four interactions where we did not have information on patient education or occupation). Of these jokes, 50 took place during consultations with patients from high SES (median jokes per consultation = 3, IQR = 2‐5), while 53 jokes were observed with patients in lower SES (median jokes per consultation = 2, IQR = 0.75‐3.25). We did not use a standardized way of defining a joke or teasing, as jokes and teasing are often a product of context, so in line with this subjective interpretation of whether jokes were made or not, we did not perform any statistical analysis.

#### 3.3.2 Small talk

In contrast, doctors seemed much colder in their interactions with lower SES patients, providing very little space for patients to speak and not attempting to initiate or encourage any sort of rapport in the same way. Physicians did much of the talking, most of which was centred around the medical aspects of the consultation with very little small talk or attempts at humour. In Table 4 (Theme 1), we give contrasting examples by patients’ SES background of small talk in both opening and farewell sequences of consultations. This shows that the discourse involving Patient 6, with a relatively low SES background, is much more focused on the functional (medical) rather than the relational aspects of the interaction, than for Patient 25, where the doctor drives the level of conversational familiarity and the patient responds appropriately.

Medical discourse is known to involve a dialectic between institutional (eg medical) frame and socio‐relational frame for talk.^25^ Thus, the consultation can be divided into task (instrumental) talk and relational (small) talk.^25^ We found that a lower proportion of the appointment was given to relational talk for low SES patients. This difference in proportion of time spent on small talk could in part be due to differences in total appointment time. Table 5 shows the mean appointment lengths for each IMD decile. Barring a few outliers, patients from less deprived areas appear to generally tend to have longer appointments, although because the sample size of this qualitative study was relatively small, it was not appropriate to use statistical tests to investigate this further.

**Table 5:** Mean appointment length by IMD decile

IMD decile	Mean appointment length (minutes: seconds) [SD]
1	7:52 [4:11]
2	6:29 [0:09]
3	6:19 [2:41]
4	7:06 [2:36]
5	13:55 [4:08]
6	5:54 [0:46]
7	7:29 [0:51]
8	15:52 [7:31]
9	9:01 [5:35]
10	7:34 [0]

### 3.4 Active or passive participation

We observed that low SES patients were comparatively passive in consultations: Physicians generally took the lead, with low SES patients raising relatively few questions and new topics of discussion. This pattern appeared to be influenced by three factors: firstly by patients’ with knowledge and experience gained in higher education or in their occupation consciously applying this to ‘oil the wheels’ of the interaction; secondly by higher SES patients coming to the consultation with their own agenda; and thirdly because lower SES saw it as the Physician's responsibility to give information—rather than it being the patient's responsibility in obtaining it.

#### 3.4.1 Education and occupation as a cultural frame of reference

Eleven patients referred to their education and occupation during interactions. This was spread evenly across SES. However, SES differences were seen in the ways in which they referenced education and occupation. More educated patients and those with insights into the workings of health services by virtue of their occupation appeared to consciously apply this knowledge to help navigate the healthcare system and to reduce the doctor‐patient difference in status. The example given in Table 4 (Theme 2, sub‐theme 2.1, Patient 12) is from an interview with a patient who previously worked as a microbiologist in the NHS, and who clearly felt his background enabled a more equal partnership; facilitating a positive interaction in the consultation for the sake of the Physician's satisfaction with the appointment, and not just their own satisfaction.

This is in contrast with a patient with lower SES (Table 3, Theme 2, sub‐theme 2.1, Patient 8) who was an auto‐electrician working on motorway signage; who also made reference to his occupation, but in a different way. Patient 8 made this reference to his occupation when talking about the extent to which he trusted the information and was reliant on the Physician's superior knowledge and expertise. This patient takes a less opinionated and more passive stance than the patient who had a more professional occupational background, in recognition of their different domains of knowledge and expertise.

#### 3.4.2 Agenda setting

In the interviews, about a third of patients (13 of 36) reported wanting specific concerns clarified in the appointment. These were evenly distributed by SES, but we found more highly educated patients appeared confident about raising concerns they had about their quality of life. Most reported asking about something at every visit, although we did not triangulate this by counting how many questions they raised during their consultation. Interview data from Patient 3 (Table 4, Theme 2, sub‐theme 2.2) illustrate this, as an example of a higher SES patient who consciously set out to make the most out of their follow‐up appointment. Higher SES patients seemed less deterred from discussing their topic of interest if initially ignored by the physician and were not concerned that they would be made to feel stupid. Pursuing a matter of concern when the Physician initially ignores the patient is illustrated here:

Physician: ‘Any problems’?

Patient: ‘Erm no, er a niggling problem. Err just like when I’m breathing, it’s like an irritation on the back of me throat like, you know like when you used to have croupe when you were a kid‐‘

Physician: ‘Mmm (writing notes during problem presentation)’.

Patient: ‘‐and you know that sort of wheezy breathe that doesn’t have anything to do with anything. It just makes me want to clear me throat all the time. (3‐second silence while Physician continues to write) I don’t know if it’s the air or you know sensitive or…’ Patient 3, Physician 2, Consultation

This was in contrast to low SES patients, who took a more passive approach as illustrated in sub‐theme 2.2 in Table 3 (Patient 7): waiting for the Physician to set the agenda which was then focused on medical, or task aspect of the appointment. Low SES patients also appeared to be relatively more reticent in raising emotional concerns as an appointment agenda item.

#### 3.4.3 Responsibility for obtaining information

When asking whether they received sufficient information, we found that higher SES patients actively sought information from the Physician, even outside of the consultation if they had forgotten to ask something during their appointment—seeing this as their responsibility: if someone has not received sufficient information then this was seen as their fault (see Patient 3, Theme 2, sub‐theme 2.3, Table 3). This contrasted with lower SES patients who saw it as the Physician's role to provide them with information which the Physician deemed to be important (see Patient 6, Table 3, Theme 2, sub‐theme 2.3).

### 3.5 Preferences for involvement

#### 3.5.1 Defining involvement in decision‐making

When patients were asked how involved they were in deciding what cancer treatment to undergo and whether they were happy with that level of involvement, we found that there were SES differences in how patients defined involvement. Lower SES patients interpreted ‘involvement’ as being fully informed of any decisions the Physician made regarding their healthcare (see Table 3, Patient 6, Theme 3, sub‐theme 3.1). They seemed uninterested in being involved in making decisions, which was also reflected our observational data: Physicians made generally decisions for them without consulting them, and they seemed happy with this.

In contrast, patients with higher education levels were more interested in being a part of the decision‐making process. Physicians also involved them in this process, talking through the various options available to them and the pros and cons of each so that these patients could make an informed decision. This is illustrated in data from Patient 3, Table 3, Theme 3, sub‐theme 3.1 where the patient talked about the conversation which took place with her Physician when she was diagnosed where both discussed two alternative treatments, one of which was undergoing a clinical trial.

#### 3.5.2 Stoicism

Stoicism is the endurance of pain or hardship without the display of feelings and without complaint.^26^ In terms of complaint, all patients brought concerns to physicians’ attention and the degree of stoicism did not appear to differ across SES. Few complained about pain or discomfort in their interviews or appointments. However, while no higher SES patients referred to their own stoicism, several low SES patients explicitly referred to stoicism as a positive and self‐defining value. This in illustrated by data from Patient 13 (Table 3, Theme 3, sub‐theme 3.2) who had an outlook on life that ‘You just have to get on with it’. This contrasted with patients with higher education levels who were keen to talk through any issues with Physicians in order to help them deal with these problems. They not only wanted to make the Physician aware of their problems but also to understand them for themselves (Patient 12, Table 3, Theme 3, sub‐theme 3.2). Low SES patients, on the other hand, appeared almost proud of delaying raising concerns or seeking help. One such patient very briefly mentioned swallowing problems in her appointment but vehemently denied it later:

Patient: ‘I am not a worrying type, if something happens in life get on with it, deal with it. That’s how I am made’. Patient 7, Physician 1, Interview

This stoicism seems to be an important part of their identity for low SES patients. One such patient spoke about it being passed down from their parents:

Patient: ‘And my mother was quite a strong woman. You know she, we were never mollycoddled as children and we were expected… They were loving parents but we were expected to err…to get on with it. As they had, they came from a different generation obviously. Which isn't a bad background to be perfectly honest, it's it's…I don’t know it spells out to you what is important in life and what’s the priorities. So I appreciated all that. And I think you do inherit some of that’. Patient 14, Physician 3, Interview

This is supported by other studies showing low SES identity includes experience of facing and surviving adversity and in this, avoiding being labelled as a victim.^27^, ^28^

## 4 DISCUSSION AND CONCLUSION

### 4.1 Discussion

In summary, firstly we found SES differences in sociable talk, that is, relational communication between Physician and patient. Although relational talk is recognized to be an important activity in itself, since the cocreation of a ‘relational climate’ promotes patient cooperation with care ^29^; our study found Physicians appeared to spend more time engaged in this, where patients were closer to their own social position. We particularly identified differences in the amount of humour within consultations. Previous research has found that individuals use humour and teasing to create power and solidarity within an interaction, with teasing particularly used in interactions between individuals of the same gender,^30^ although since our study involved only consultations with male Physicians we recognize that there may be gender differences, possibly in line with gender concordance between doctor and patient, and so further studies of this would be helpful.

Our finding that patients from less deprived areas had longer appointments is consistent with the findings from our previous study which also showed a significant positive correlation between appointment length and SES as measured by IMD decile.^31^ This may reflect the lack of much relational talk which occurred between Physicians and low SES patients. Coupland et al^32^ report that both patients and doctors work to sustain the relational portion of the consultation which can delay the instrumental portion.^32^ However, in our study we found that the relational portion appeared relatively reduced in consultations involving low SES patients, and instead, there was a prioritizing of the instrumental aspects of the consultation. Because of the qualitative nature of the study, with a limited sample size, we did not use statistical methods to investigate differences in relational talk by socioeconomic gradient, and so further (quantitative) studies would be needed to investigate the pattern which emerged in the data.

The finding from the sub‐theme ‘stoicism’ that patients with lower education levels seemed to prefer to deal with problems on their own in private as a way of coping with their condition is supported by previous research. For example, a study conducted with breast cancer patients found that low SES patients raised significantly fewer concerns during consultations with their oncologist.^9^ Some studies have found that individuals from low SES backgrounds tend to expect negative outcomes, which can lead to hopelessness and chronic stress.^33^ They engage in more fatalism and avoidance, as opposed to instrumental coping behaviours such as talking through issues with their doctor, displaying lower perceived control over events.^34^, ^35^ This is in line with the SES differences in the patterns of doctor‐patient communication we observed in our study too.

Within the ‘Defining involvement in decision‐making’ theme, we also identified that patients with less education were not only less likely to be involved in making decisions about their care, but that they defined involvement as being fully informed of decisions made rather than making decisions themselves. They seemed content with this. For example, a low SES patient explained: ‘…I expect to be 100% involved…You know if, say he was going to operate on me or things like that…I would like to be involved. You know I would want to know everything about it before it actually happened…’ (Table 3). Although previous studies which have also found that people from low SES backgrounds are less likely to seek information or have a preference for active involvement in decision‐making,^36^, ^37^, ^38^ few have identified that SES differences in the patients’ preferences for participation may contribute to this. Our finding that low SES patients defined involvement as being fully informed of decisions which the Physician made is reflected in other studies findings.^39^, ^40^ For example, a Swedish study of frail elderly patients found that patients had a similar definition of ‘involvement’ (wanting information from healthcare staff but still taking a relatively passive role).^39^ This finding was in relation to age differences however, and so our study highlights that these differences are pertinent to differences by SES too.

There are two systematic reviews which have looked at SES differences in doctor‐patient communication, finding that low SES patients both receive more directive consultations from doctors, but also participate less actively themselves.^8^, ^41^ Currently, these conclude that doctors provide low SES patients with less information because they assume that such patients do not want as much information; however, our findings challenge this. Our study suggests that these doctors may be correct in their assumption that patients across the socioeconomic gradient prioritize different aspects of their care, resulting in differing behaviours, preferences and levels of participation. Patients and doctors can have different perceptions of the communication behaviours which are utilized by the doctor in the interaction^42^; however, in the context of our findings, this disagreement does not necessarily mean dissatisfaction on the part of the patient. Since low SES patients take a more passive approach to information exchange, they may be satisfied with not being encouraged to ask questions in the consultation.

Nevertheless, increasing patients’ control over their own healthcare is increasingly seen as a good thing,^43^ and something which contributes to better treatment outcomes.^1^, ^2^, ^3^, ^4^, ^5^ Therefore, even if not driven by the need to satisfy low SES patients’ expectations, there may still be a need to ensure that information exchange during consultations is more equitable by socioeconomic status. That our previous study found no SES differences in the number of concerns raised in head and neck cancer clinics when using a question prompt list^31^ suggests that a pro forma approach might be an effective intervention to reduce socioeconomic inequalities in this area. The Patient Concerns Inventory (PCI) is a question prompt list which allows patients to select what topics they wish to discuss with their Physician, prior to their appointment. This is then used to streamline the consultation.^44^ Studies have found that it is feasible to use with both elderly patients and those with little education,^45^, ^46^ and currently, a trial is being conducted to examine whether its long‐term use may improve patients’ quality of life.^47^

While patients’ control over their own healthcare has increased over time,^43^ researchers and clinicians should be mindful that not all patients are interested in taking a more active role in their care.

Our study has a number of limitations. Firstly, our observation of the consultations may have altered the way in which they were conducted. Both patients and Physicians may have acted differently than they usually would have, because they were aware of being observed and recorded. In some consultations, references were made by both patients and Physicians to the recording equipment being used, so this is a possibility, although one which many qualitative studies of this nature experience. We could have also used video‐recording equipment to collect more data from the consultations themselves, however, that may have potentially influenced participants’ behaviours even more.

It is also important to acknowledge the impact of our own expectations and experiences on the analysis. The researcher grew up in a lower/middle class household, but has since received training as a healthcare professional and researcher and objectively sits in a higher SES bracket. These experiences are likely to have led to unconscious biases within the analysis.

## 5 CONCLUSION

In conclusion, there are a number of socioeconomic differences in communication behaviours during head and neck oncology follow‐up consultations. This is through the use of relational talk, patients’ active or passive participation, and patients’ preferences for involvement. Patients from low SES backgrounds seem to take a passive role within the consultation, which may cause the Physician to alter their communication style accordingly.

### 5.1 Practice implications

Given that patients from lower socioeconomic backgrounds seem to be reluctant to raise concerns with Physicians, the PCI may be a useful way to facilitate raising of concerns for such patients. This may help to reduce SES differences in this aspect of doctor‐patient communication, and potentially reduce health inequalities in this group.

## CONFLICTS OF INTEREST

None.

## Data Availability

Research data are not shared.

## References

[1] Fors A , Gyllensten H , Swedberg K , Ekman I . Effectiveness of person‐centred care after acute coronary syndrome in relation to educational level: Subgroup analysis of a two‐armed randomised controlled trial. Int J Cardiol. 2016;221:957‐962. 27441475 10.1016/j.ijcard.2016.07.060

[2] Jani B , Bikker AP , Higgins M , et al. Patient centredness and the outcome of primary care consultations with patients with depression in areas of high and low socioeconomic deprivation, Article. Br J General Pract. 2012;62(601):2. 10.3399/bjgp12X653633 PMC3404336 22867682

[3] Mead N , Bower P . Patient‐centred consultations and outcomes in primary care: a review of the literature. Patient Educ Counsel. 2002;48(1):51‐61. 10.1016/s0738-3991(02)00099-x 12220750

[4] Stewart MA . Effective physician‐patient communication and health outcomes ‐ a review. Review. Can Med Assoc J. 1995;152(9):1423‐1433. 7728691 PMC1337906

[5] Pirhonen L , Olofsson EH , Fors A , Ekman I , Bolin K . Effects of person‐centred care on health outcomes—A randomized controlled trial in patients with acute coronary syndrome. Health Policy. 2017;121(2):169‐179. 28062091 10.1016/j.healthpol.2016.12.003

[6] Ha JF , Longnecker N . Doctor‐patient communication: a review. The Ochsner Journal. 2010;10(1):38‐43. 21603354 PMC3096184

[7] Epstein RM , Street JRRL . Patient‐centered communication in cancer care: promoting healing and reducing suffering. 2007.

[8] Verlinde E , De Laender N , De Maesschalck S , Deveugele M , Willems S . The social gradient in doctor‐patient communication. Int J Equity Health. 2012;11(1):12. 22409902 10.1186/1475-9276-11-12 PMC3317830

[9] Siminoff LA , Graham GC , Gordon NH . Cancer communication patterns and the influence of patient characteristics: disparities in information‐giving and affective behaviors. Patient Educ Couns. 2006;62(3):355‐360. 16860520 10.1016/j.pec.2006.06.011

[10] Hall JA , Roter DL , Katz NR . Meta‐analysis of correlates of provider behavior in medical encounters. Med Care. 1988;26(7):657‐675. 3292851 10.1097/00005650-198807000-00002

[11] Martin E , Russell D , Goodwin S , Chapman R , North M , Sheridan PWHY . Patients consult and what happens when they do. Article. Br Med J. 1991;303(6797):289‐292. 1888932 10.1136/bmj.303.6797.289 PMC1670469

[12] Berger J , Rosenholtz SJ , Zelditch M Jr . Status organizing processes. Ann Rev Sociol. 1980;6(1):479‐508.

[13] Peck BM , Conner S . Talking with me or talking at me? the impact of status characteristics on doctor‐patient interaction. Article. Soc Perspectives 2011;54(4):547‐567.

[14] Tan JJX , Kraus MW . Lay theories about social class buffer lower‐class individuals against poor self‐rated health and negative affect. Article. Personality Soc Psychol Bulletin. 2015;41(3):446‐461. 10.1177/0146167215569705 25634909

[15] Allen S , Rogers SN , Harris RV . Socio‐economic differences in patient participation behaviours in doctor–patient interactions—A systematic mapping review of the literature. Health Expect. 2019;22(5):1173‐1184. 31398772 10.1111/hex.12956 PMC6803421

[16] Cr UK. https://www.cancerresearchuk.org/health-professional/cancer-statistics/statistics-by-cancer-type/head-and-neck-cancers#heading-Zero. Accessed September 10, 2020

[17] Rylands J , Lowe D , Rogers SN . Influence of deprivation on health‐related quality of life of patients with cancer of the head and neck in Merseyside and Cheshire. Br J Oral Maxillofacial Surg. 2016;54(6):669‐676. 10.1016/j.bjoms.2016.03.030 27130568

[18] Rylands J , Lowe D , Rogers SN . Outcomes by area of residence deprivation in a cohort of oral cancer patients: survival, health‐related quality of life, and place of death. Oral Oncol. 2016;52:30‐36. 26586367 10.1016/j.oraloncology.2015.10.017

[19] The English Indices of Deprivation. 2015.

[20] Coelho HF , Murray L , Royal‐Lawson M , Cooper PJ . Antenatal anxiety disorder as a predictor of postnatal depression: a longitudinal study. J Affect Dis. 2011;129(1‐3):348‐353. 20805004 10.1016/j.jad.2010.08.002

[21] Tagiyeva N , Semple S , Devereux G , et al. Reconstructing past occupational exposures: how reliable are women's reports of their partner's occupation? Occup Environ Med. 2011;68(6):452‐456. 21098830 10.1136/oem.2009.052506

[22] Krapohl E , Plomin R . Genetic link between family socioeconomic status and children’s educational achievement estimated from genome‐wide SNPs. Mol Psychiatry. 2016;21(3):437‐443. 25754083 10.1038/mp.2015.2 PMC4486001

[23] Classification SO . Standard Occupational Classification Volume 1: Structure & Descriptions of Unit Group. Office for National Statistics. London: The Stationery Office; 2020.

[24] Classification SO . Standard Occupational Classification Volume 2: The Coding Index and Coding Rules and Conventions. Office for National Statistics. London: The Stationery Office; 2020.

[25] Ragan SL . Sociable talk in women's health care contexts: two forms of non‐medical talk. Small Talk. Routledge 2014;289‐307.

[26] Wagstaff GF , Rowledge AM . Stoicism: its relation to gender, attitudes toward poverty, and reactions to emotive material. J Soc Psychol. 1995;135(2):181‐184. 7776642 10.1080/00224545.1995.9711421

[27] Bolam B , Murphy S , Gleeson K . Individualisation and inequalities in health: a qualitative study of class identity and health. Soc Sci Med. 2004;59(7):1355‐1365. 15246166 10.1016/j.socscimed.2004.01.018

[28] Bolam B , Hodgetts D , Chamberlain K , Murphy S , Gleeson K . 'Just do it': an analysis of accounts of control over health amongst lower socioeconomic status groups. Crit Public Health. 2003;13(1):15‐31.

[29] Coupland J . Small Talk. London: Routledge; 2014.

[30] Hay J . Functions of humor in the conversations of men and women. J Pragmat. 2000;32(6):709‐742.

[31] Allen S , Harris R , Brown SL , Humphris G , Zhou Y , Rogers SN . High levels of socioeconomic deprivation do not inhibit patients' communication of concerns in head and neck cancer review clinics. Review. Br J Oral Maxillofac Surg. 2018;56(6):536‐539. 29908705 10.1016/j.bjoms.2018.05.015

[32] Coupland J , Robinson JD , Coupland N . Frame negotiation in doctor‐elderly patient consultations. Discourse Soc. 1994;5(1):89‐124.

[33] Kristenson M , Eriksen HR , Sluiter JK , Starke D , Ursin H . Psychobiological mechanisms of socioeconomic differences in health. Review. Soc Sci Med. 2004;58(8):1511‐1522. 14759694 10.1016/S0277-9536(03)00353-8

[34] Caplan LJ , Schooler C . Socioeconomic status and financial coping strategies: the mediating role of perceived control. Soc Psychol Quart. 2007;70(1):43‐58.

[35] Westbrook MT . Socioeconomic differences in coping with childbearing. Am J Community Psychol. 1979;7(4):397‐412. 495582 10.1007/BF00894382

[36] Arora NK , McHorney CA . Patient preferences for medical decision making: who really wants to participate? Med Care. 2000;38(3):335‐341. 10718358 10.1097/00005650-200003000-00010

[37] Garfield S , Smith F , Francis SA , et al. Can patients' preferences for involvement in decision‐making regarding the use of medicines be predicted? Patient Educ Couns. 2007;66(3):361‐367. 17331691 10.1016/j.pec.2007.01.012

[38] Lee CJ , Ramirez AS , Lewis N , Gray SW , Hornik RC . Looking beyond the internet: examining socioeconomic inequalities in cancer information seeking among cancer patients. Article. Health Commun. 2012;27(8):806‐817. 22356137 10.1080/10410236.2011.647621 PMC4209720

[39] Ekdahl AW , Andersson L , Friedrichsen M . "They do what they think is the best for me." Frail elderly patients' preferences for participation in their care during hospitalization. Article. Patient Educ Couns. 2010;80(2):233‐240. 19945814 10.1016/j.pec.2009.10.026

[40] Avis M . Choice cuts ‐ an exploratory‐study of patients views about participation in decision‐making in a day surgery unit. Article. Int J Nurs Stud. 1994;31(3):289‐298. 8088941 10.1016/0020-7489(94)90055-8

[41] Willems S , De Maesschalck S , Deveugele M , Derese A , DeMaeseneer J Socio‐economic status of the patient and doctor–patient communication: does it make a difference? Patient Educ Counsel. 2005;56(2):139‐146. 10.1016/j.pec.2004.02.011 15653242

[42] Kenny DA , Veldhuijzen W , Tvd W , et al. Interpersonal perception in the context of doctor–patient relationships: a dyadic analysis of doctor–patient communication. Soc Sci Med. 2010;70(5):763‐768. 20005618 10.1016/j.socscimed.2009.10.065

[43] Harrison N . Regressing or progressing: what next for the doctor–patient relationship? Lancet Respir Med. 2018;6(3):178‐180. 29508703 10.1016/S2213-2600(18)30075-4

[44] Rogers SN , Lowe D . An evaluation of the Head and neck cancer patient concerns inventory across the merseyside and cheshire network. Article. Br J Oral Maxillofac Surg. 2014;52(7):615‐623. 24927654 10.1016/j.bjoms.2014.04.011

[45] Hatta JMM , Doss JG , Rogers SN . The feasibility of using Patients Concerns Inventory (PCI) in managing Malaysian oral cancer patients. Article. Int J Oral Maxillofac Surg. 2014;43(2):147‐155. 24074487 10.1016/j.ijom.2013.08.006

[46] Rogers SN , Audisio RA , Lowe D . Do the elderly raise different issues when using the Patient Concerns Inventory in routine head and neck cancer follow‐up clinics? Article. Eur J Cancer Care. 2015;24(2):189‐197. 10.1111/ecc.12289 25651100

[47] Rogers S , Lowe D , Lowies C , et al. Improving quality of life through routine use of the Patient Concerns Inventory (PCI) for head and neck cancer patients: a cluster preference randomized controlled trial. Meeting Abstract. Psycho‐Oncology. 2019;28:10‐11. 10.1186/s12885-018-4355-0 PMC5907378 29669529

